# Gaint phyllodes tumour with axillary & interpectoral lymph node metastasis; A rare presentation

**DOI:** 10.1016/j.ijscr.2019.12.026

**Published:** 2019-12-26

**Authors:** Alam Ara Shafi, Bandar AlHarthi, Muhammad Masood Riaz, Asim AlBagir

**Affiliations:** a Department of Surgical Oncology, King Fahad Medical City, Riyadh, Saudi Arabia; b Department of General Surgery, North West Anglia Trust UK

**Keywords:** Phyllodes tumour, Axillary lymph node metastasis, Breast surgery

## Abstract

**Introduction:**

Phyllodes tumors are rare fibroepithelial neoplasms of the breast, account for less than 1 % of all breast neoplasms, lymph node metastasis is even rare and routine axillary dissection is not recommended. Approximately 20 % of patients with malignant phyllodes tumors develop distant metastasis.

**Case Description:**

A 45 years old premenopausal female, presented with a huge right breast mass for 3 years. Physical examination revealed a 40 × 46 cm, lobulated mass, fixed to the chest wall and multiple enlarged and mobile ipsilateral axillary lymphadenopathy. Core tissue biopsy revealed fibroepithelial & stromal proliferation suggestive of phyllodes tumor.

The CT scan of the chest and the abdomen revealed bilateral pulmonary metastasis.

She underwent palliative Modified Radical Mastectomy. Pathology demonstrated malignant phyllodes with osseous and chondroid metaplasia, the resection margins were clear and 6 out of 25 nodes were positive.

Her post-operative recovery was uneventful and she received adjuvant chemo-radiotherapy. During 12 months follow up, no local recurrence was noted, but despite chemotherapy her pulmonary disease was increasing.

**Discussion:**

Only a few cases of cystosarcoma phyllodes with lymph node involvement have been reported in the literature. Treves, Norris and Taylor’s series have demonstrated the axillary node metastasis of less that 1 %. Since most sarcomas metastasize hematogenously, this finding explains why axillary metastasis is so rare. Hence most authors have concluded that removal of axillary lymph nodes is not warranted unless there are pathologically involved.

**Conclusion:**

Management of Phyllodes tumor presents the surgeon with challenges. Core tissue biopsy is a reliable method for pre-operative diagnosis. Imaging like CT scan and MRI help to evaluate the primary lesion as well as distant metastasis. The majority of these cases can be managed by simple mastectomy. Axillary lymph node metastasis is rare and dissection should be limited to patients with pathological evidence of tumor in the lymph nodes.

## Highlights

• Phyllodes tumors of the breast are rare biphasic fibroepithelial neoplasms that account for less than 1 % of all breast neoplasms.
• Metastasis to the Axillary nodes is an extremely rare phenomenon and clearly predicts the prognosis of the disease.
• A formal axillary lymph node dissection should not be a routine practice, rather to be limited to the patients with pathological evidence of tumor in lymph nodes.

## 1 Introduction

Phyllodes tumors are rare mesenchymal tumors with a diverse biologic behavior. They account for less than 1 % of all breast neoplasms [1]. First describe by Johanes Muller in 1838, he coined the term cystosarcoma phyllodes; a misleading description as the tumor are rarely cystic and majority of them follow a benign clinical course. WHO adopted the term phyllodes tumors in 1981 and these are now sub classified as benign, borderline and malignant based upon stromal characteristics [2]. Triple assessment by clinical, radiological and histological examination forms the fundamental basis for the evaluation of all breast lumps [3,4]. Phyllodes tumor should be considered in women, particularly over 35 years, who present with rapidly growing breast lump. Treatment of choice is radical excision in the form of lumpectomy or mastectomy, with negative margins. The nodal metastasis is rare and routine axillary dissection is not recommended. Generally 10–40 % of these tumors take malignant course with a higher tendency of local recurrence and systemic metastasis. Approximately 20–30 % of patients with malignant phyllodes tumor develop distant metastasis, spreading hematogenously to lungs (60 %), bones (28 %) and brain (9 %) [5]. The role of chemotherapy, radiotherapy and hormonal manipulation in both the adjuvant and palliative settings remains a matter of debate.

This work has been reported in line with the SCARE criteria. [6]

## 2 Case presentation

A 45 years old female, presented with 3 years history of huge right breast mass. She noticed rapid growth about four months prior to the presentation. There was no personal or family history of breast cancer. Her past medical history was insignificant. Her menarche was at the age of 13 and she had five children.

**Fig. 1:**
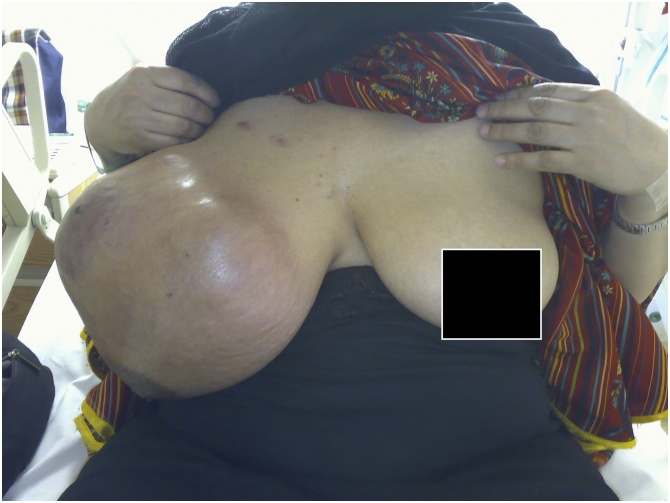
Phyllodes tumor right breast (Front view).

**Fig. 2:**
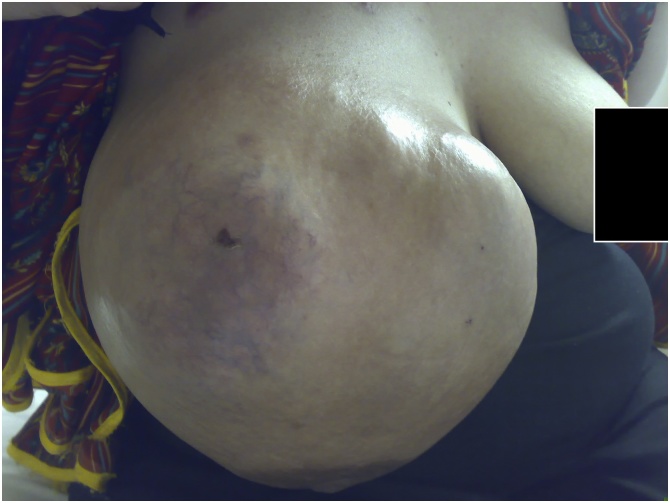
Phyllodes tumor right breast (Lateral view).

Physical examination revealed a mass measuring 40 × 46 cm, lobulated and cystic-solid in consistency, fixed to the chest wall and overlying erythematus skin with prominent veins and normal nipple areola complex [Fig. 1, Fig. 2]. Multiple enlarged and mobile lymph nodes were palpable in the right axilla; the contra-lateral breast, axilla and neck examination was normal.

Breast ultrasound showed a heterogeneous mass occupying the right breast with solid and cystic components, having fluid collections and multiple enlarged right axillary lymph nodes.

Core tissue biopsy revealed prominent mixed fibroepithelial and stromal proliferation, suggestive of phyllodes tumor.

**Fig. 3:**
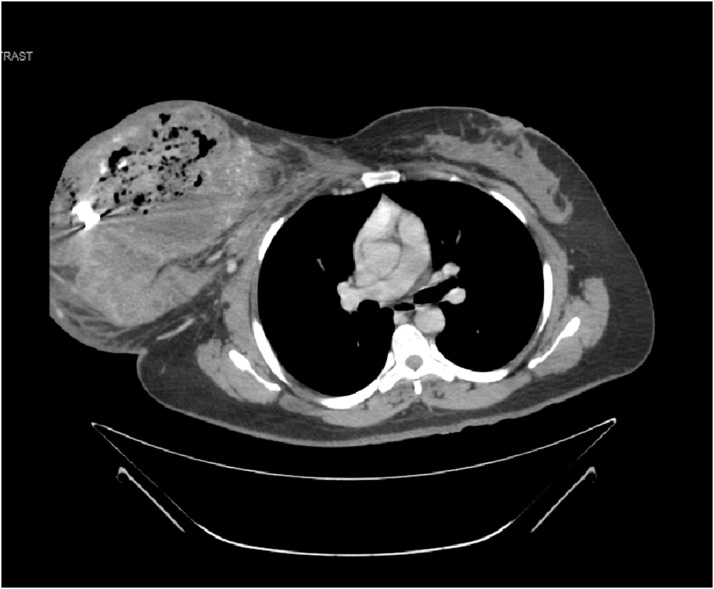
CT-Scan chest Transvers section showing huge heterogeneous right breast mass.

**Fig. 4:**
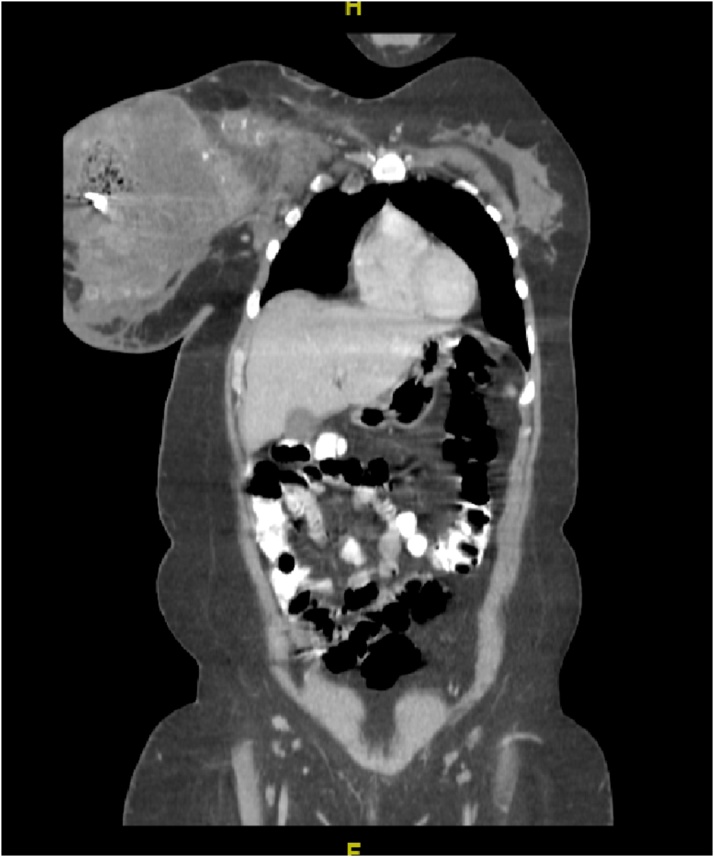
CT-Scan chest coronal section.

CT scan of the chest and the abdomen revealed; bilateral pulmonary metastasis without mediastinal adenopathy, invasion of tumor into the chest wall, axillary lymph node enlargement with central necrosis and an enlarged hyper vascular inter-pectoral lymph node measuring 2.5 × 2.5 cm [Fig. 3, Fig. 4].

She was diagnosed as metastatic phylloides tumor and underwent palliative modified radical mastectomy for her symptoms. The involved pectorals major muscle was excised as en-bloc, the enlarged inter-pectoral lymph node was excised separately and the wound was closed primarily.

**Fig. 5:**
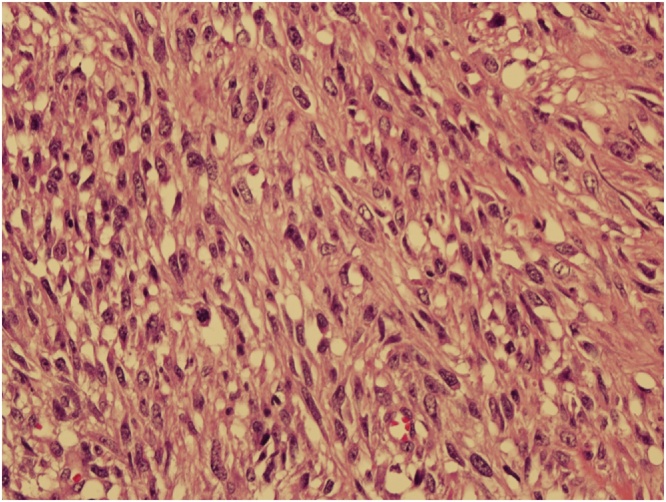
High power view-marked stromal overgrowth of spindle cells, cellular polymorphism and high mitotic figures.

**Fig. 6:**
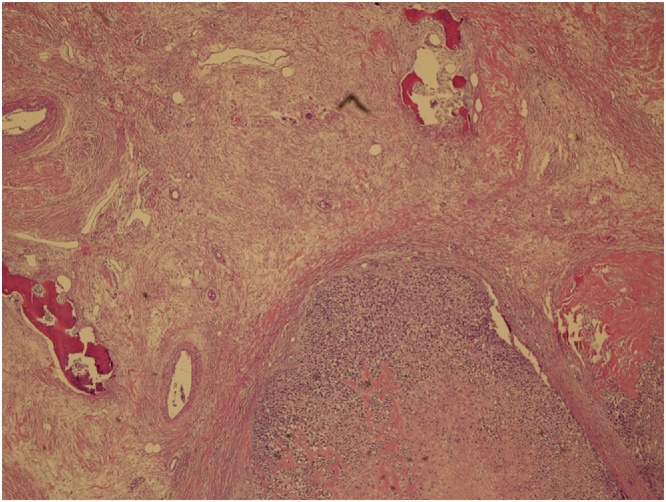
The Phyllodes with osseous and chondroid metaplasia.

**Fig. 7:**
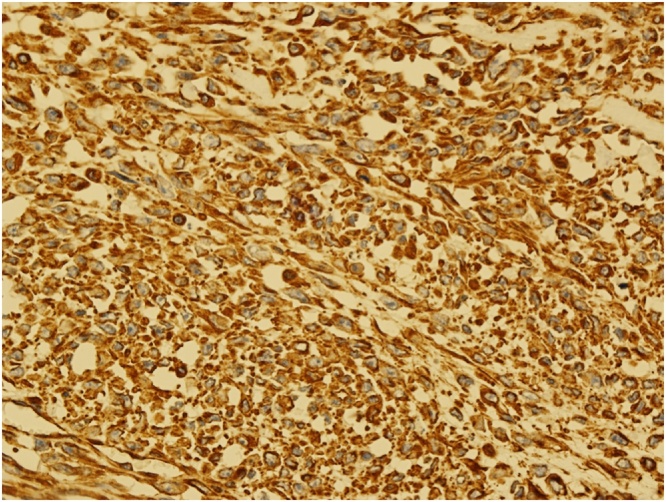
Vimentin immunostain positive tumor cells.

**Fig. 8:**
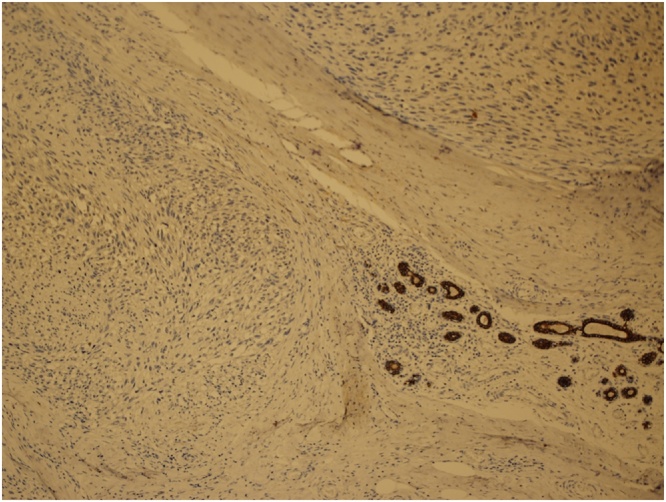
Cytokeratin immunostain positive benign breast tissue-the tumor cells are negative.

**Fig. 9:**
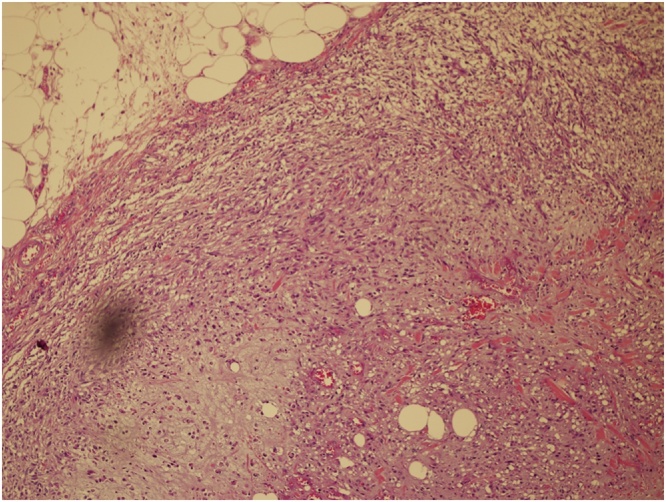
Lymph node metastasis.

Grossly the specimen measured 35 × 39 cm with nipple areola complex measuring 6 × 5 cm, focal ulcerated area also identified measuring 3 × 5 cm and axillary tail 13 × 7 cm ex vivo. Additionally, grossly identified enlarged axillary lymph node measured 5 × 5 cm. The histopathological findings were consistent with malignant phylloides tumor with osseous and chondroid metaplasia (Fig. 5, Fig. 6). The resection margins were free of tumor. Tumor was positive for Vimentin and negative for Cytokeratin immunostains (Fig. 7, Fig. 8). Six out of 25 axillary nodes were positive with perinodal extension, the largest one measuring 5 cm in diameter. The interpectoral node was also found to be positive for metastasis and measured 3 × 2.5 cm (Fig. 9).

**Fig. 10:**
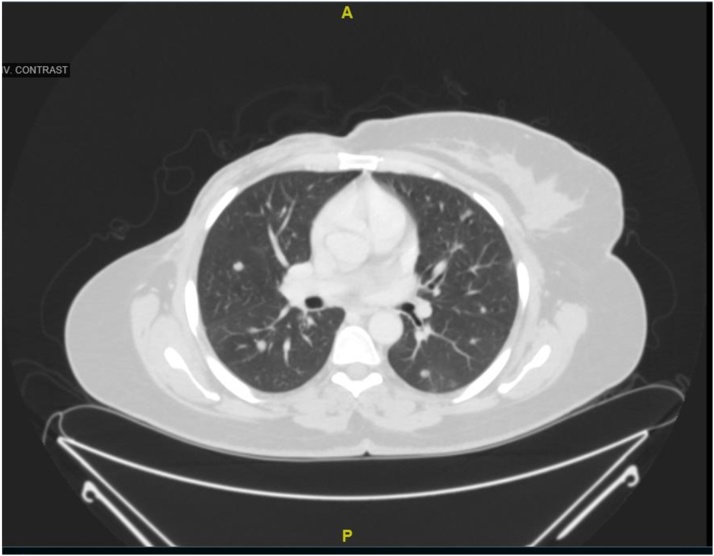
CT-Scan chest demonstrating bilateral multiple pulmonary nodules (Post-operative).

Her post-operative recovery was uneventful with well healed wound. She was referred to medical oncology where she received chemo-radiotherapy. During her post operative follow up for 12 months, no local recurrence was noted but her pulmonary nodules were increasing in number and size despite chemotherapy (Fig. 10).

## 3 Discussion

Phylloides tumors are fibroepithelial neoplasms comprise of epithelial and cellular stromal components. The presence of both these elements is necessary to confirm the diagnosis. Although, stroma is the neoplastic component and determines the pathological behavior [7,8]. The incidence is about 2.1 per million, the peak occurs in women aged 45–49 years [9,10].

Classically, patients present with a painless, firm, mobile, rounded or macrolobulated, rapidly growing mas [3]. The lesion has been apparent for several years with clinical presentation precipitated by a sudden increase in size [11,12]. Malignant tumors are often larger and faster growing.

There are no definitive mammographic or ultrasound features for phyllodes per se, which make it extremely difficult to differentiate from a fibroadenoma. Rowel et al. and Chua et al, in a series of 106 patients demonstrated that 71 % of patients with a post-operative diagnosis of phyllodes tumor had a presumptive diagnosis of fibroadenoma preoperatively [9,12]. Early diagnosis of phyllodes tumor leads to accurate and timely management, including surgery; consequently enormous growth of the tumor into giant ones, like our case, be prevented.

A variety of techniques have been utilized to improve the preoperative diagnosis of phyllodes tumor. No ultrasonic and mammographic indicators have been identified that allow differentiation between benign and malignant tumors [13]. Cole-Beuglet et al. performed a retrospective study on 8 cases of histopathologically proven phyllodes tumors that were evaluated by mammography and ultrasound. They determined that while certain ultrasound findings (low-level internal echoes, smooth walls, good through transmission, and smooth margined fluid-filled clefts in a predominantly solid mass) may suggest a phyllodes tumor, there is no consistent and reliable way to distinguish between phyllodes tumors and other benign appearing tumors on ultrasound or mammography [14].

Reinfuss et al., using histotype criteria developed by Azzopardi and Salvalori et al., [15] showed that the histotype of the tumor was an independent prognostic factor, with 5-year survivals of 95.7 % for benign tumors, 73.7 % for borderline tumors, and 66.1 % for malignant tumors [16]. A review and clinical follow-up of 33 cases concluded that histopathological classification is the strongest prognostic factor [17]. Metastasis occurs hematogenously to the lungs (66 %), bones (28 %), and brain (9 %) and, in rare instances, to the liver and heart [15]. Regional lymph node enlargement due to metastasis is a rare phenomenon [18].

Only a few cases of cystosarcoma phyllodes with lymph node involvement have been reported in the literature. Treves, in his series of 33 cases, reported only 1 case that showed metastasis to the axillary lymph nodes [19]. In Norris and Taylor’s series of 94 patients, 16 (17 %) had enlarged lymph nodes, but only 1 (1 %) patient had histologically proven metastasis [20]. Reinfuss et al. found axillary lymphadenopathy clinically in 11 (20 %) of 55 patients, but only 1 (1.8 %) showed metastasis [21]. Staren et al. found axillary lymph node involvement in 1 out of 26 patients [22]. Thus, the incidence of axillary lymph node involvement in these series ranged from 1.1%–3.8%. Since most sarcomas metastasize hematogenously, this finding may explain why axillary metastasis is so rare. Observing the rarity of lymph node involvement, most authors have concluded that removal of axillary lymph nodes is not warranted unless there are palpable [16,23]. Data regarding sentinel lymph node biopsy in phyllodes tumors are lacking. Our patient had palpable axillary lymphadenopathy, so she underwent axillary dissection.

Staging of phyllodes tumors follows the guidelines for sarcoma [24]. Because of the rarity of these cases and the variety of therapies utilized, it is difficult to extrapolate data and use the information to treat individual patients. Treatment of malignant phyllodes tumors is based upon retrospective reviews in the literature.

Surgical therapy is aimed at complete excision of the tumor with at least 1-cm margins in the form of either lumpectomy or mastectomy, depending upon the tumor size to the breast ratio. Mastectomy has not shown a survival advantage over wide local excision [21]. The strongest independent predictor of local recurrence is a positive margin. Chaney et al., described a subset of eight patients with phyllodes tumors who received adjuvant radiation for one or more indications, including malignant tumors, tumors larger than 10 cm, or a positive margin [25]. One patient had a previous recurrence. No patient had metastatic disease at presentation. Radiotherapy of 7000 cGy was administered to the chest wall/breast of the eight patients. No local or distant failures were seen at a median follow-up of 36.5 months. The use of radiotherapy remains controversial, as other retrospective studies have failed to prove any benefit [21]. Doxorubicin-based adjuvant chemotherapy is recommended for breast sarcomas' first-line treatment [26].

## 4 Conclusion

The phyllodes tumor management presents the surgeon with challenges. Core tissue biopsy is considered a reliable method for pre-operative diagnosis while radiological imaging like CT scan and MRI help to evaluate the primary lesion as well as distant metastasis. Main bulk of these cases can be managed by simple mastectomy and axillary dissection should be limited to the patients with pathological evidence of tumor in the lymph nodes. Skin graft and immediate reconstruction with myocutaneous flap remain the options for wide defects after mastectomy.

## Declaration of Competing Interest

We don’t have any conflicts of interest with any person, organisation or institution.

## Funding

No funding was granted from any organisation or institution.

## Ethical approval

This case report got exempted from ethical committee

## Author contribution

Study conception and design: Shafi

Acquisition of data: AlBagir

Analysis and interpretation of data: Shafi

Drafting of manuscript: Shafi and Riaz

Critical revision: AlHarthi

## Research studies

Registration is not required for the case reports.

## Guarantor

Alam Ara Shafi

## Provenance and peer review

Not commissioned, externally peer-reviewed
